# Microbiome, Autoimmune Diseases and HIV Infection: Friends or Foes?

**DOI:** 10.3390/nu11112629

**Published:** 2019-11-02

**Authors:** Chiara Pellicano, Giorgia Leodori, Giuseppe Pietro Innocenti, Antonietta Gigante, Edoardo Rosato

**Affiliations:** 1 Department of Translational and Precision Medicine-Scleroderma Unit, Sapienza University of Rome, 00185 Rome, Italy; chiara.pellicano@uniroma1.it (C.P.); giorgia.leodori@uniroma1.it (G.L.); antonietta.gigante@uniroma1.it (A.G.); 2 Department of Public Health and Infectious Diseases, Sapienza University of Rome, 00185 Rome Italy; giuseppe.innocenti@uniroma1.it

**Keywords:** microbiota, autoimmune diseases, HIV, eubiosis, dysbiosis

## Abstract

Several studies highlighted the importance of the interaction between microbiota and the immune system in the development and maintenance of the homeostasis of the human organism. Dysbiosis is associated with proinflammatory and pathological state-like metabolic diseases, autoimmune diseases and HIV infection. In this review, we discuss the current understanding of the possible role of dysbiosis in triggering and/or exacerbating symptoms of autoimmune diseases and HIV infection. There are no data about the influence of the microbiome on the development of autoimmune diseases during HIV infection. We can hypothesize that untreated patients may be more susceptible to the development of autoimmune diseases, due to the presence of dysbiosis. Eubiosis, re-established by probiotic administration, can be used to reduce triggers for autoimmune diseases in untreated HIV patients, although clinical studies are needed to evaluate the role of the microbiome in autoimmune diseases in HIV patients.

## 1. Introduction

The human microbiome is the aggregate of all microbiota, encompassing bacteria, archaea, fungi, protists and symbionts, that reside on or within human tissues and biofluids [1]. The species of bacteria that are most characterized in the microbiome, conversely, are the viruses and fungi. The characterization of the microbiome was primarily made by cell culture. Recently the microbiome was evaluated by DNA recombinant techniques [1]. Several studies highlighted the importance of the interaction between microbiota and the immune system in the development and maintenance of the homeostasis of the human organism [2,3,4]. The microbiome is influenced by diet [5,6], aging [6,7,8] and lifestyle and changes during human life in the type and number of bacteria [7]. The greatest conditioning factor for gut microbiota composition, diversity and richness seems to be the diet [5]. Over time, eating habits have changed—the diet is poor in carbohydrates and fibers and rich in fat and sugar and this has altered the balance of the bacterial population [5,9]. It has been seen that a diet rich in animal fats allows the development of *Bacteroides*, while a diet rich in carbohydrates or plant-based foods and low in animal fats favors the growth of *Prevotella* [5]. However, high *Prevotella* has also been described in inflammatory states such as rheumatoid arthritis (RA) [10] and has been linked with obesity [11] and insulin resistance [12]. During eubiosis, a healthy and balanced state marked by high diversity and the abundance of microbial populations, gut microbiota is mainly composed of *Actinobacteria*, *Bacteroidetes*, *Proteobacteria* and *Firmicutes*. Most of these are crucial for the synthesis of vitamins, the degradation of xenobiotics and sterols and the deconjugation of biliary acids [13]. Furthermore, microbiota can inhibit pathogens’ epithelial adhesion, colonization and translocation, competing for adhesion sites and nutrients, releasing bacteriocins and stimulating neutrophil migration and functions, IgA secretion and T cells maturation and differentiation [13,14,15,16,17,18]. Short-chain fatty acids (SCFAs), mainly butyrate, acetate and propionate, are produced by gut microbiota through the fermentation of fibers. They represent a source of energy for colonocytes and promote cellular reparation and differentiation, protecting the integrity of the intestinal barrier [19,20,21]. Moreover, SCFAs promote the differentiation of T cells into T regulatory (Treg) cells and inhibit inflammasome activation, leading to a tolerogenic phenotype, through the modifications of histone deacetylase and by the activation of metabolite-sensing G proteins-coupled receptors [22,23,24,25].

Recent studies have shown that SCFAs can promote the development of some autoimmune diseases, favoring the release of interleukins (i.e., IL10) and reducing the inflammatory response [26,27,28], metabolic diseases and neurological diseases [29]. In particular, SCFAs are able to modulate immune cell chemotaxis, reactive oxygen species (ROS) release as well as cytokine release. Zhang et al. showed an improvement of gastrointestinal symptoms in a mice model of colitis after the administration of butyrate by increasing Treg cells [30]. Treg cells are abundant in the lamina propria of the gut and microbiota might modulate their functions through IL10 and TGFbeta production, reducing inappropriate inflammatory responses [31,32]. It has been demonstrated that tryptophan-derived metabolites, produced by gut microbiota and the active aryl hydrocarbon receptor (AHR) on innate lymphoid cells (ILCs), induce the production of IL22 that provides colonization resistance to *Candida albicans* [33,34]. It has been hypothesized that there is a possible association between any alteration of the human microbiome (dysbiosis) and several diseases. Dysbiosis is associated with proinflammatory and pathological state-like obesity [11], HIV infection and such autoimmune diseases as Type 1 diabetes (T1D) [35,36,37,38,39,40,41,42,43,44,45,46,47,48,49,50,51,52,53,54,55,56,57,58,59,60], RA [61,62,63,64,65,66,67,68,69], systemic lupus erythematosus (SLE) [70,71,72,73,74], Sjögren’s syndrome (SS) [75,76,77,78], systemic sclerosis (SSc) [79,80], inflammatory bowel disease (IBD) [81,82,83,84,85,86,87,88,89,90,91], coeliac disease [92], autoimmune liver diseases [93,94,95,96,97,98,99,100,101,102,103], Behcet’s disease (BD) [104,105,106,107] and psoriasis vulgaris [108,109,110,111,112,113]. Dysbiosis, induced by several environmental factors (i.e., virus infections, drugs, diet), alters the fragile balance between microbiota and host, so there may be the development of autoimmune disease [2,114]. Table 1 summarizes the main alterations of the gut microbiota, found in the course of autoimmune diseases and HIV infection, and the main underlying pathogenetic mechanisms.

## 2. Type 1 Diabetes

T1D is a systemic and chronic disease due to the marked and progressive inability of the pancreas to secrete insulin because of the autoimmune destruction of the beta cells, which results in an alteration of carbohydrate, fat and protein metabolism [128]. Currently, autoimmunity is considered the major factor in the pathophysiology of T1D. In a genetically susceptible individual, viral infection may stimulate the production of antibodies against a viral protein that trigger an autoimmune response against antigenically similar beta cell molecules. Several studies on animal models [36,37,38,39,40,44,51] and humans [42,43,45,46,48,49,53] have hypothesized a critical role of gut dysbiosis in T1D onset [47], as well as diet [50]. The possible role of molecular mimicry by the microbiome in immune tolerance breakdown is supported by the evidence of a microbial peptide mimic of the islet-specific glucose-6-phosphatase catalytic subunit-related protein (IGRP), an islet-specific auto-antigen, in accelerated T1D MyD88-/- mice with an altered intestinal microbiome [129]. The Old Friends Hypothesis sustains that dysbiosis, which results in a loss of commensal microbes evolved together with their host, might have a role in the host’s immune response regulation and homeostasis [130]. Probably, a combination of dysbiosis, increased gut permeability and an impaired intestinal immune responsiveness intrude together, leading to anti-islet autoimmunity, as the Perfect Storm Hypothesis postulates [131]. Maffeis et al. observed in Italian T1D-affected children an increased intestinal permeability that correlates with alterations in the microbiota composition with an increase in *Dialister invisus*, *Globicatella sanguinis* and *Bifidobacterium longum* in T1D patients as compared to healthy controls [115]. Huang et al. found a prevalence of *Bacteroidetes* in gut microbiota of 12 T1D Han Chinese children. Conversely, Firmicutes were prevalent in healthy controls [57], according to a previous study conducted on Caucasian patients [50]. These data support the hypothesis of reduced epithelial barrier activity due to alterations of epithelial tight junctions caused by products of *Bacteroidetes* anaerobic respiration (i.e., acetate and succinate) [50].

## 3. Rheumatoid Arthritis

RA is a chronic systemic inflammatory disease. In genetically susceptible individuals, an autoimmune reaction, triggered by environmental factors, leads to synovial hypertrophy and chronic joint inflammation, along with the potential for extra-articular manifestations [132]. The microbiome may have a pivotal role in the development of autoimmunity as suggested by the observation that germ-free mice were protected against experimental arthritis [117,133]. It has been hypothesized an important contribution of segmented filamentous bacteria (SFB) is in the development of autoimmune arthritis, influencing adaptive and innate immunity through enhanced Th17 infiltration in the intestinal lamina propria [117,133,134,135,136,137]. Moreover, SFB might selectively expand Th17 cells expressing dual TCRs, which recognize both SFB antigens and self-antigens. These cells are recruited to the lung by CCL20-CCR6 axis and trigger RA-related lung autoimmunity [138]. An alteration of the gut microbiome in RA patients is described—new-onset RA patients have a higher abundance of *Prevotella copri* than patients with established RA [63,139]. The theory of molecular mimicry in RA is supported by the evidence of two auto-antigens (*N*-acetylglucosamine-6-sulfatase (GNS) and filamin A (FLNA)) with sequence homology to epitopes of *Prevotella* spp. [65]. These self-antigens are expressed in inflamed synovial tissues and GNS antibody values correlate with anti-citrullinated protein antibodies (ACPAs) [65]. High levels of ACPAs are associated with periodontitis, suggesting a role of infection by *Porphyromonas gingivalis* and RA onset [140]. It has been proposed that the citrullination of peptides by peptidylarginine deiminase (PAD), an enzyme expressed by *P. gingivalis*, might break immune tolerance [116]. Furthermore, RA patients present a greater alteration in gut microbial diversity than controls, with a reduction of *Faecalibacterium* and an increase of *Lactobacillaceae* and *Bacilli* [65,68,69].

## 4. Systemic Lupus Erythematosus

SLE is a chronic inflammatory disease with a highly variable clinical presentation and course [141]. Although a correlation between SLE development and dysbiosis has not been demonstrated, several studies observed an alteration of the microbiome composition with an increase of the *Bacteroides* phyla and *Lachnospiraceae* and a decrease in the *Firmicutes* and *Lactobacillaceae* [72,118]. According to Johnson et al., dysbiosis is associated with local inflammatory responses (specifically the Th17 response) and high circulating levels of antibodies against ds-DNA and histone [117]. In addition, the possible role of periodontal disease in the SLE condition has been investigated [70,71,73], as has been demonstrated by the alteration of subgingival microbiota, with a more elevated subgingival bacterial load and a reduced microbial diversity at the diseased sites than in controls [74]. In the literature, there are no exhaustive data on the possible influence of the microbiome and its alterations in the development of the antiphospholipid antibody syndrome, both primary and secondary to LES.

## 5. Sjögren’s Syndrome

SS is a systemic chronic inflammatory disorder characterized by lymphocytic infiltrates in exocrine organs and the presence of specific antibodies against Ro (SS-A) and La (SS-B) antigens [142]. A possible role in the pathogenesis of SS has been hypothesized for the microbiome since peptides derived from oral, gut and skin commensal bacteria (*P. disiens*, *Capnocytophaga* spp., *B. finegoldii*, *B. intestinalis*, *B. fragilis*, *Alistipes finegoldii*, *Corynebacterium amycolatum* and *Acinetobacter johnsonii*) may induce an immune response by the activation of Ro60-reactive T cells [75,76,77,78]. Furthermore, Mandl et al. found higher disease activity (evaluated by Sjögren’s Syndrome Disease Activity Index), lower levels of complement component and higher levels of fecal calprotectin in patients with decreased levels of *Bifidobacterium* and *Alisipes* [78].

## 6. Systemic Sclerosis

SSc is a systemic connective tissue disease, characterized by the aberrant activation of the immune system, functional and structural vascular damage and the progressive fibrosis of skin and visceral organs [143]. Most patients have dysfunction of the gastrointestinal tract and alteration of the composition of the gut microbiota with low levels of *Faecalibacterium* and *Clostridium* and high levels of *Fusobacterium*, *γ-Proteobacteria*, *Bifidobacterium* and *Lactobacillus* [79,80]. Moreover, a reduction of *Faecalibacterium prausnitzii* and *Clostridiaceae* and an increase of *Lactobacillus* were more pronounced among patients with esophageal dysfunction and malnutrition [80]. It has been shown that gut microbiota alterations, due to proton pump inhibitors (PPI) use favors *C. difficile* infection [144]. In particular, the chronic use of PPI causes dysbiosis with an increase in *Bacteroidetes* compared to *Firmicutes*, which favors the development of enteric infections [120].

## 7. Inflammatory Bowel Diseases

Ulcerative colitis (UC) is a diffuse, nonspecific inflammatory disease whose etiology is unknown [145]. A variety of immunologic changes have been documented in UC Subsets of T cells accumulate in the lamina propria of the diseased colonic segment and these T cells are cytotoxic to the colonic epithelium. This change is accompanied by an increase in the population of B cells and plasma cells, with an increased production of immunoglobulin G (IgG) and immunoglobulin E (IgE) [146]. A small proportion of patients with UC have antismooth muscle (ASMA) and anticytoskeletal antibodies. Serum and mucosal autoantibodies against intestinal epithelial cells may be involved in the alteration of the intestinal epithelial barrier. The presence of antineutrophil cytoplasmic antibodies (ANCA) and anti–Saccharomyces cerevisiae antibodies (ASCA) is a feature of inflammatory bowel disease [147]. Sulfate-reducing bacteria, which produce sulfides, are found in large numbers in patients with UC, and sulfide production is higher in patients with UC than in other people. Sulfide production is even higher in patients with active UC than in patients in remission and a decrease in Klebsiella species is seen in the ileum of patients relative to control subjects. This difference disappears after proctocolectomy [148]. Crohn disease (CD) is an idiopathic, chronic regional enteritis that most commonly affects the terminal ileum but has the potential to affect any part of the gastrointestinal tract from mouth to anus. A combination of factors, including aberrant mucosal immune responses, intestinal epithelial dysfunction and defects of host interactions with intestinal microbes, likely contribute to CD [149]. The alteration of innate and adaptive immune responses directed towards pathogen-associated molecular patterns (PAMPs) derived from intestinal microbiota in genetically susceptible patients contributes to the pathogenesis of these diseases. This hypothesis is supported by the observation of impaired epithelial barriers and increased intestinal permeability in UC and CD patients [121]. Several studies reported a correlation between dysbiosis and IBD and it could be present before the onset of the disease [81,83,84,90]. An increase in the Proteobacteria has been observed in patients with UC or CD, whereas Firmicutes was reduced in the fecal samples of CD patients with respect to healthy individuals [82,83,86]. Furthermore, there is a reduction in butyrate-producing bacteria (i.e., *Bacteroides*, *Eubacterium*, *Faecalibacterium* and *Ruminococcus*) [87,89].

## 8. Coeliac Disease

Coeliac disease is an inflammatory disease of the small intestine, triggered by wheat gliadin (gluten) [150]. A reduction of *Streptococcus mutans* and *Streptococcus anginosus* has been seen in patients with coeliac disease, compared to healthy people. Moreover, there is an increase of *Staphylococcus epidermidis*, *Staphylococcus pasteuri* and *Klebsiella oxytoca* in duodenal biopsies of coeliac patients [92]. Dysbiosis may have a role in the onset and/or progression of coeliac disease, though further studies are needed to elucidate this assumption.

## 9. Autoimmune Liver Diseases

Primary sclerosing cholangitis (PSC) is a chronic liver disease characterized by a progressive course of cholestasis with inflammation and fibrosis of the intrahepatic and extrahepatic bile ducts [151]. The condition may lead to cirrhosis of the liver with portal hypertension and end-stage liver disease [151]. An autoimmune mechanism is suggested because approximately 75%–90% of patients with PSC have IBD, however, only approximately 4% of patients with IBD have or develop PSC [152]. A marked increase in serum autoantibody levels occurs in patients with PSC as well, with ANCA in 87%, anticardiolipin (aCL) antibodies in 66% and antinuclear antibodies (ANA) in 53%. It has been reported that PSC and IBD have overlapping yet distinct genetic architectures [153]. Furthermore, in the biliary ducts, an inflammatory response to chronic or recurrent bacterial infection in the portal circulation and from exposure to toxic bile acids has been postulated [154]. Primary biliary cholangitis (PBC) is a chronic disease of the liver, possibly autoimmune in nature, which leads to progressive cholestasis and often end-stage liver disease [155]. It is characterized by abnormalities of the humoral and cellular immune systems (i.e., elevated serum levels of immunoglobulins), circulating autoantibodies (i.e., antimitochondrial antibodies, AMAs), granulomas in the liver and regional lymph nodes, the impaired regulation of both B and T lymphocytes and the association with a variety of autoimmune-mediated diseases (i.e., autoimmune thyroiditis, SSc, SS) [155]. Subsequent to the loss of the intrahepatic bile ducts, mediated by activated CD4 and CD8 lymphocytes, a disruption of the normal bile flow occurs with the retention and deposition of toxic substances, with a further secondary destruction of the bile ducts and the hepatocytes. In addition, increased expression of the HLA class II antigens in the liver occurs, rendering the hepatocytes and bile duct epithelial cells more susceptible to activated T lymphocytes and perhaps exacerbating immunologically mediated cytotoxicity [122]. Moreover, infection with organisms of the family *Enterobacteriaceae* may play a role in the pathogenesis of this disease. It has been postulated that a cross-reactivity between antigens on the bacterial wall and the mitochondria, as bacterial lipoteichoic acid is detected around the damaged bile ducts in PBC and chronic bacterial exposure in normal mice, leads to autoantigen production and subsequent cholangitis that mimics PBC [156]. Intestinal dysbiosis has been associated with these diseases and several studies have reported an altered gut microbial community in PSC and PBC, although there are discrepancies at species levels [93,94,95,96,97,98,99,100,101,102,103].

## 10. Behcet’s Disease

BD is a rare vasculitic disorder characterized by a triple-symptom complex of recurrent oral aphthous ulcers, genital ulcers and uveitis, though systemic manifestations can be heterogeneous [157]. The specific etiology of BD remains elusive but the interplay between genetic factors and infectious-agent exposure may have a role in eliciting a cross-reactive immune response responsible for the vascular damage [157]. Several studies highlighted the atypical composition of the gut microbiome in BD patients [104,105,106,107] Ye et al. showed an increase of *Bilophila* spp. and several opportunistic pathogens (i.e., *Parabacteroides* spp. and *Paraprevotella* spp.) and a reduction in butyrate-producing bacteria, *Clostridium* spp. and methanogens (*Methanoculleus* spp. and *Methanomethylophilus* spp.) in fecal samples from active BD patients [107]. Moreover, the fecal microbiota transplant from active BD patients in B10RIII mice exacerbated experimental autoimmune uveitis activity, with strong inflammatory cell infiltration within the retina, the choroid and the vitreous cavity [107]. The possible association between BD and specific gut microbiome alterations is supported by the demonstration that BD patients show defects in Th1, Th17 and Treg cells, whose functions are regulated by gut microbiota [123,158,159].

## 11. Psoriasis

Psoriasis is a chronic, multifactorial, inflammatory disease that involves the hyperproliferation of the keratinocytes in the epidermis, with an increase in the epidermal cell turnover rate. The epidermis is infiltrated by a large number of activated T cells, which appear to be capable of inducing keratinocyte proliferation. T cell hyperactivity and the resulting proinflammatory mediators (IL17/23) play a major role in the pathogenesis of psoriasis [160]. Chang et al. confirmed a marked upregulation of the Th17 response, which could have a role in IL17-driven inflammation in psoriasis [112]. A statistically significant association between psoriasis and IBD and another important comorbidity of psoriasis is psoriatic arthritis has been demonstrated [161,162]. The gut microbiota profile of patients with psoriatic arthritis and patients with psoriasis showed decreased bacterial diversity and a reduced abundance of *Akkermansia*, *Ruminococcus* and *Pseudobutyrivibrio* compared to healthy controls, and the microbiota profile of psoriatic arthritis resembled that published for patients with IBD [113].

## 12. HIV Infection

The human immunodeficiency virus (HIV) is a retrovirus. HIV binds to receptors of CD4 and to the coreceptor CCR5. This link favors the entry of the virus into T-helper lymphocyte and viral replication. The course of the disease is divided into phases based on clinical changes and CD4 cell count. The last phase is the acquired immunodeficiency syndrome (AIDS), characterized by opportunistic infections and tumors. The introduction of highly active antiretroviral therapy (HAART) has allowed an improvement in the life expectancy of HIV-positive patients [163,164,165]. However, HAART treatment does not protect against an increased risk of infectious diseases and non-infectious chronic comorbidities, typical of HIV-positive individuals [165,166]. Several studies have characterized the microbiome of subjects with HIV. There are no unique results in the literature about the microbiome in HIV patients. Most of the studies reported a change in bacterial gut composition [124,125,126,127,167,168]. Current data indicate increases in commensal bacteria that may be pathogenic in HIV patients, and decreases in beneficial commensals [125,169,170]. The proportion of the families of bacteria change in HIV patients, but not the diversity of these [169]. The proportion of *Firmicutes/Bacteroidetes* seems to increase significantly in HIV-1-infected patients because the concentration of *Bacteroidetes* decreases and that of *Prevotella* increases [124,169,171]. A significant difference in concentration and in the types of SCFAs was highlighted in HIV-positive patients compared to HIV-negative subjects. In HIV-positive patients, propionic acid increased and acetic and butyric acid had a significantly lower concentration [125,169,171]. The relationship with the use of HAART and the microbiota of HIV patients is unclear [172]. A possible hypothesis is that HIV therapy promotes the restoration of normal microbial flora [173]. Other studies show a minimal role of HAART [126,127,173] or even its negative impact [168]. Synthesizing these data, both HIV and antiviral therapy seem to play a different role in the type of microbiome. These studies show a strong correlation between HIV and dysbiosis. The presence of one (HIV) favors the development of the other (dysbiosis) and vice versa [170].

## 13. Autoimmune Disease and HIV Infection

The immune alteration (depending on the CD4 and CD8 count) may lead to the development of autoimmune diseases, although this is not a frequent event [8]. Rheumatological diseases such as reactive arthritis, psoriatic arthritis and various forms of connective tissue diseases are less frequent after the introduction of HAART among HIV-infected individuals [11,12]. Some authors have hypothesized that autoimmune diseases may develop in the first stage of infection, when the symptoms of HIV are few and CD4 count is still high [166].

In the HAART era, the thrombocytopenia (ITP) is the main autoimmune disease associated to HIV [174]. It is an autoimmune coagulation disorder characterized by isolated thrombocytopenia (platelets < 100,000/microL) and causes severe visceral bleeding (hematuria, gastrointestinal or cerebro-meningeal haemorrhage). In the early stage of HIV infection, HIV-related ITP depends on immune-mediated peripheral platelet destruction (anti-GpIIIa antibodies and immune complexes). In the late phase of HIV infection, there is a defect of platelet production [175,176]. Sarcoidosis is a multisystemic disease, the causes of which are not known, which is characterized by the formation of immune granulomas in the organs. In granulomas, CD4 lymphocytes accumulate and for this reason, sarcoidosis is rare in patients with AIDS [177]. Polymyositis (PM) is associated with HIV through an unknown mechanism. While dermatomyositis (DM) is rarer, PM can occur in all stages of HIV infection regardless of the state of immunodeficiency. It presents with classical proximal weakness, myalgia, mechanic’s hands, dactylitis and possible interstitial lung disease, and it is important to distinguish this form from the toxic effect of drugs (i.e., zidovudine, stavudine), which improves with the end of HAART treatment [178]. In the literature, there are conflicting data regarding the association between autoimmune thyroid diseases and HIV infection [179,180]. The association with HIV infection and other autoimmune diseases such as RA, autoimmune liver diseases and SLE is quite rare [8].

## 14. Role of Probiotics and Fecal Microbiota Transplantation

Eubiosis can be re-established by probiotic (mainly *Bifidobacterium* spp. and *Lactobacillus* spp.) administration or by fecal microbiota transplantation (FMT) [181,182,183]. Several studies showed a reduction of proinflammatory cytokines (IL1, IL6, TNFalfa, IL12, IL17) and an increase in IL10 production in RA patients after the administration of *Lactobacillus casei*, *L. acidophilus* and *Bifidobacterium bifidum*, associated with improvement in the disease activity score [184]. In the literature, there are less data about the possible benefit of FMT in patients with autoimmune diseases, although it is considered a rescue therapy in patients with IBD [185].

## 15. Conclusions

In the literature, there are no data about the influence of the microbiome on the development of autoimmune diseases during HIV infection. However, we can hypothesize that untreated patients may be more susceptible to the development of autoimmune diseases, due to the presence of dysbiosis. We can suppose that CD4+ T lymphocytes depletion in HIV-infected and untreated patients results in CD8 T lymphocyte and B lymphocyte hyperfunction or expansion. CD40-activated B cells are potent antigen-presenting cells that induce specific T cell responses in vitro and in vivo. The CD40-activated B cell was able to induce an autoimmune response by the activation of CD8+ T lymphocytes. We can therefore hypothesize that the activation of APC with CD8+ T lymphocytes recruitment (i.e., IBD, psoriasis and ankylosing spondylitis) can facilitate the development of autoimmune diseases. Eubiosis, re-established by probiotic administration, can be used to reduce triggers for autoimmune diseases in untreated HIV patients. Clinical studies are needed to evaluate the role of the microbiome in autoimmune diseases in HIV patients.

## Figures and Tables

**Table 1:** Main alterations of gut microbiota and underlying pathogenetic mechanisms.

Disease	Microbiota Alteration	Mechanism	References
Type 1 Diabetes Mellitus	↑ *Bacteroidetes/Firmicutes* ratio, *D. invisus*, *G. sanguinis*, *B. longum* ↓ *B. adolescentis*, *Lactobacillus*	↑ intestinal permeability, ↓ Treg differentiation due to ↓ SCFA	Maffeis et al. [115], Huang et al. [57], Mejia-Leon et al. [50]
Rheumatoid Arthritis	↑ *P. copri*, *P. gingivalis*, *Bacilli* and *Lactobacillales* ↓ *Faecalibacterium*	Molecular mimicry (FLNA, GNS), ↑ citrullinated proteins and Th17 pathway	Zhang et al. [63], Pianta et al. [65], Scher et al. [113], Montgomery et al. [116], Wu et al. [117]
Systemic Lupus Erythematosus	↑ *Bacteroidetes/Firmicutes* ratio ↓ *Lactobacillaceae*	↑ Th17	Hevia et al. [72], Zhang et al. [118], Johnson et al. [119]
Sjögren’s Syndrome	↑ *B. intestinalis*, *B. fragilis*	↑ activation of Ro60-reactive T cells, molecular mimicry	Szymula et al. [75], De Paiva et al. [77]
Systemic Sclerosis	↑ *Bacteroidetes/Firmicutes* ratio	↑ esophageal dysfunction, PPI use	Volkmann et al. [79], Andreasson et al. [80], Clooney et al. [120]
Inflammatory Bowel Diseases	↑ *Proteobacteria* ↓ *Firmicutes*, *Bacteroides*, *Eubacterium*, *Faecalibacterium*, *Ruminococcus*	↑ intestinal permeability, ↓ Treg differentiation due to ↓ SCFA and AHR agonists	Manichanh et al. [83], Wang et al. [87], Takahashi et al. [89], Geremia et al. [121]
Coeliac Disease	↑ *S. mutans*, *S. anginosus*, *S. epidermidis*, *S. pasteuri*, *K. oxytoca*	↑ dysbiosis	Nagao-Kitamoto et al. [92]
Autoimmune Liver Diseases	↑ *Veillonella*, *Enterobacteriaceae*, *Enterococcus*, *Lactobacillus*	Molecular mimicry, ↑ ursodeoxycholic acid	Tang et al. [93], Hov et al. [95], Kummen et al. [102], Ma et al. [122]
Behcet’s Disease	↑ *Bilophila*, *Parabacteroides*, *Paraprevotella* ↓ butyrate-producing bacteria, *Clostridium* spp. and methanogens	↓ Treg differentiation due to ↓ SCFA	Ye et al. [107], Tanabe et al. [123]
Psoriasis	↓ *Akkermansia*, *Ruminococcus*, *Pseudobutyrivibrio*	↑ Th17	Chang et al. [112], Scher et al. [113]
HIV Infection	↓ *Lactobacillales*, *Bacteroidetes*, *Bifidobacteria*, ↑ *Pseudomonas*, *Streptococcus*, *Candida*, *Proteobacteria*, *Prevotella*, *Enterobacteriales*	Sexual habits, GI barrier dysfunction, viral load, CD4+ count	Dillon et al. [124], Mutlu et al. [125], Lozupone CA [126,127]

SCFAs: Short-chain fatty acids, FLNA: Filamin A, GNS: *N*-acetylglucosamine-6-sulfatase, PPI: Proton pump inhibitors, AHR: Active aryl hydrocarbon receptor, GI: Gastrointestinal, HIV: Human immunodeficiency virus. ↑ = increase, ↓ = decrease.
